# Recent Advances in Osteoclast Biological Behavior

**DOI:** 10.3389/fcell.2021.788680

**Published:** 2021-12-08

**Authors:** Yang Sun, Jiangbi Li, Xiaoping Xie, Feng Gu, Zhenjiang Sui, Ke Zhang, Tiecheng Yu

**Affiliations:** Department of Orthopedics, The First Hospital of Jilin University, Changchun, China

**Keywords:** osteoclast, origin, differentiation, apoptosis, functions, intercellular interactions

## Abstract

With the progress of the aging population, bone-related diseases such as osteoporosis and osteoarthritis have become urgent problems. Recent studies have demonstrated the importance of osteoclasts in bone homeostasis, implying these will be an important mediator in the treatment of bone-related diseases. Up to now, several reviews have been performed on part of osteoclast biological behaviors such as differentiation, function, or apoptosis. However, few reviews have shown the complete osteoclast biology and research advances in recent years. Therefore, in this review, we focus on the origin, differentiation, apoptosis, behavior changes and coupling signals with osteoblasts, providing a simple but comprehensive overview of osteoclasts for subsequent studies.

## 1 Introduction

The human skeleton is a complicated structure that aids mobility, controls calcium levels in blood, supports soft tissue and organ, and is a place for adult hematopoiesis. Continuous remodeling is necessary to maintain these critical capabilities by minimizing bone damage accumulation and preserving both bone mechanical strength and calcium balance (Clarke, 2008; Feng and McDonald, 2011). Remodeling of the bone is a tightly coupled process involving osteoclasts and osteoblasts. And osteoclasts are the body is only bone-resorbing cells, and they are essential for bone remodeling.

Osteoclasts are multinucleated cells created by the fusion of osteoclast progenitor cells (OCPs) with the capacity to dissolve bone matrix *via* secreting H+, Cl−, cathepsin K (CTSK), and matrix metalloproteinases (MMPs) in the resorption zone in response to macrophage colony stimulating factor (M-CSF) and receptor activator of NFkB ligand (RANKL).In addition, the differentiation and activity of osteoclasts are closely regulated by apoptosis and molecules produced by cross-talk between osteoclasts and osteoblasts, which contribute to homeostatic conditions in bone. While proper modulation ensures normal function of the skeleton, an imbalance may result in both postmenopausal and secondary forms of osteoporosis like diabetes-associated and glucocorticoid-induced osteoporosis (Albright, 1940).

With the improvement of molecular and genetic tools, our comprehension of osteoclast biology has evolved significantly. Several reviews have been performed on part of osteoclasts biological behaviors such as differentiation, function, or apoptosis (Teitelbaum and Ross, 2003; Rubin and Greenfield, 2005). However, few reviews have shown the complete osteoclast biology and recent advancements in studies. In this chapter, we collected the latest findings on osteoclast to provide references for follow-up studies.

## 2 The Differentiation of Osteoclasts

### 2.1 The Differentiation Process of Osteoclasts

**FIGURE 1:**
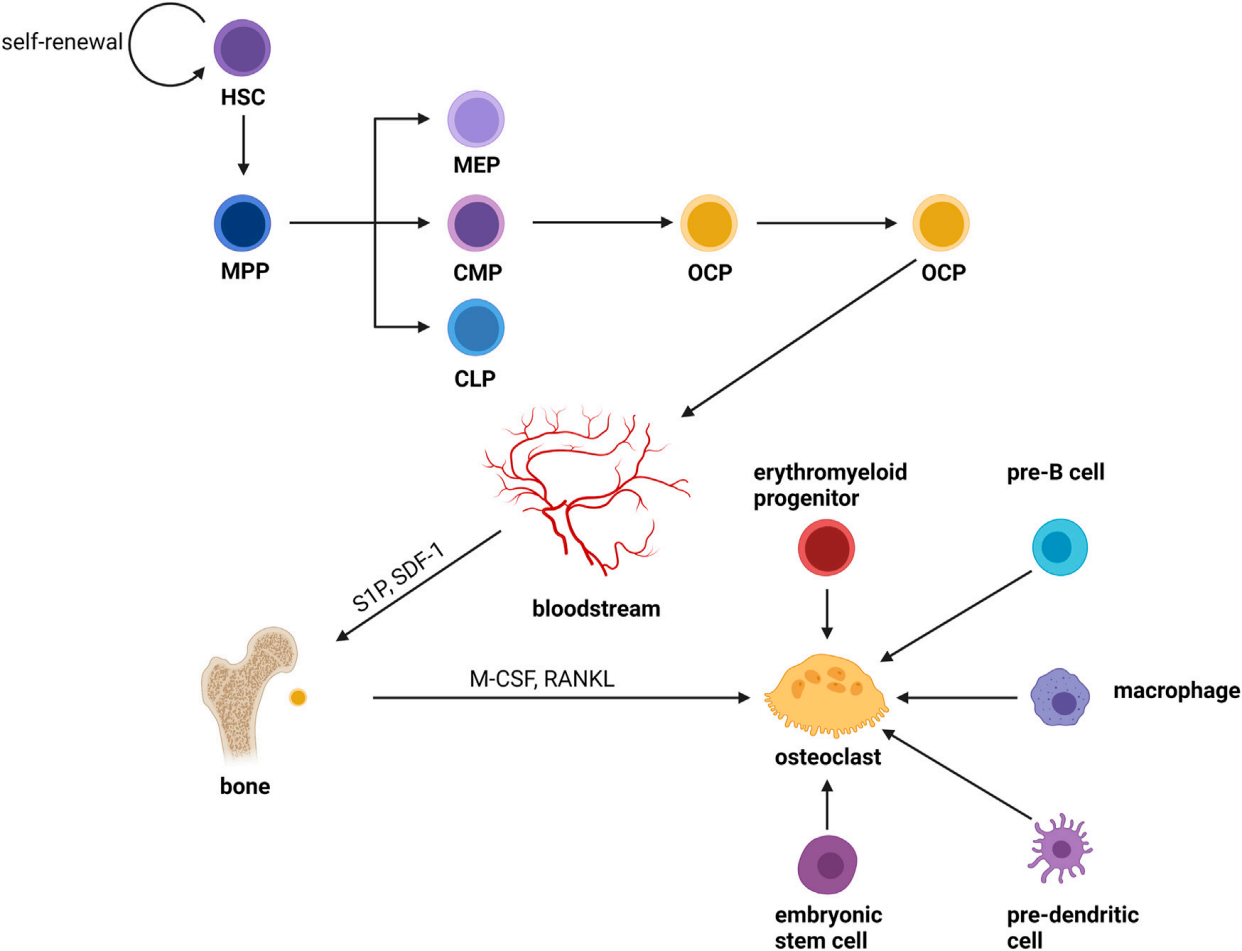
Osteoclast differentiation. Osteoclasts are formed by the fusion of OCPs from the monocyte/macrophage lineage of the bone marrow which is produced from hematopoietic stem cells (HSCs). HSCs do not form mature cells directly; instead, they yield a variety of oligopotent progenitor cells, which then differentiate into lineage-specific progenitor cells, building a hierarchy branching tree. HSCs drop their ability to self-renew and transform into multipotent progenitors (MPPs) with pluripotency. After a series of divergences, MPPs become oligopotent progenitors, which include common myeloid progenitors (CMPs), megakaryocyte–erythrocyte progenitors (MEPs), and common lymphoid progenitors (CLPs). Pre-monocytes originate from CMPs and further develop into OCPs. Then, OCPs enter the blood circulation and recruit to bone remodeling units (BRUs) under the action of factors like sphingosine-1-phosphate (S1P) and stroma-derived factor 1 (SDF-1). Finally, the mature osteoclasts were formed by the cellular fusion of OCPs in the BRUs in the stimulation of factors such as M-CSF and RANKL. Furthermore, in the inflammatory and immunological environment, monocytes or tissue-specific macrophages (macrophages that dwell in tissues) are an important source of osteoclasts. In the fetus, erythromyeloid progenitors are responsible for forming osteoclasts involved in hematopoiesis and fracture repair. Previous studies had shown that primary cells like embryonic stem cells, pre-B cells and pre-dendritic cells can also form osteoclasts.

Osteoclasts are formed by the fusion of OCPs from the monocyte/macrophage lineage of the bone marrow which is produced from hematopoietic stem cells (HSCs) (Udagawa et al., 1990). HSCs do not form mature cells directly; instead, they yield a variety of oligopotent progenitor cells, which then differentiate into lineage-specific progenitor cells, building a hierarchy branching tree. HSCs drop their ability to self-renew and transform into multipotent progenitors (MPPs) with pluripotency (Morrison and Weissman, 1994; Christensen and Weissman, 2001). After a series of cellular differentiation, MPPs become oligopotent progenitors, which include common myeloid progenitors (CMPs) (Akashi et al., 2000), megakaryocyte–erythrocyte progenitors (MEPs) (Pronk et al., 2007), and common lymphoid progenitors (CLPs) (Kondo et al., 1997; Karsunky et al., 2008; Serwold et al., 2009). Pre-monocytes originate from CMPs and further develop into OCPs. Then, OCPs enter the blood circulation and recruit to bone remodeling units (BRUs) under the action of factors like sphingosine-1-phosphate (S1P) (Kikuta et al., 2011) and stroma-derived factor 1 (SDF-1) (Zhang et al., 2008). Finally, the mature osteoclasts are formed by the cellular fusion of OCPs in the BRUs in the stimulation of factors such as M-CSF and RANKL. Furthermore, in the inflammatory and immunological environment, monocytes or tissue-specific macrophages (macrophages that dwell in tissues) are an important source of osteoclasts (Yao et al., 2021). In the fetus, erythromyeloid progenitors are responsible for forming osteoclasts involved in hematopoiesis and fracture repair (Jacome-Galarza et al., 2019; Yahara et al., 2020) Previous studies had shown that primary cells like embryonic stem cells, pre-B cells and pre-dendritic cells can also form osteoclasts (Suda et al., 1999; Speziani et al., 2007; Gomez Perdiguero et al., 2015; Chakraborty et al., 2018; Khass et al., 2019; Krishnamurthy et al., 2019) (Figure 1). The physiological mechanism is unknown, but it shows that the source of osteoclasts is not single.

### 2.2 Factors Affecting Osteoclast Differentiation

The differentiation process of osteoclasts is stimulated by a variety of signal molecules. However, the two most critical factors, M-CSF and RANKL, run through the entire process of osteoclast differentiation (Robling et al., 2006).

**FIGURE 2:**
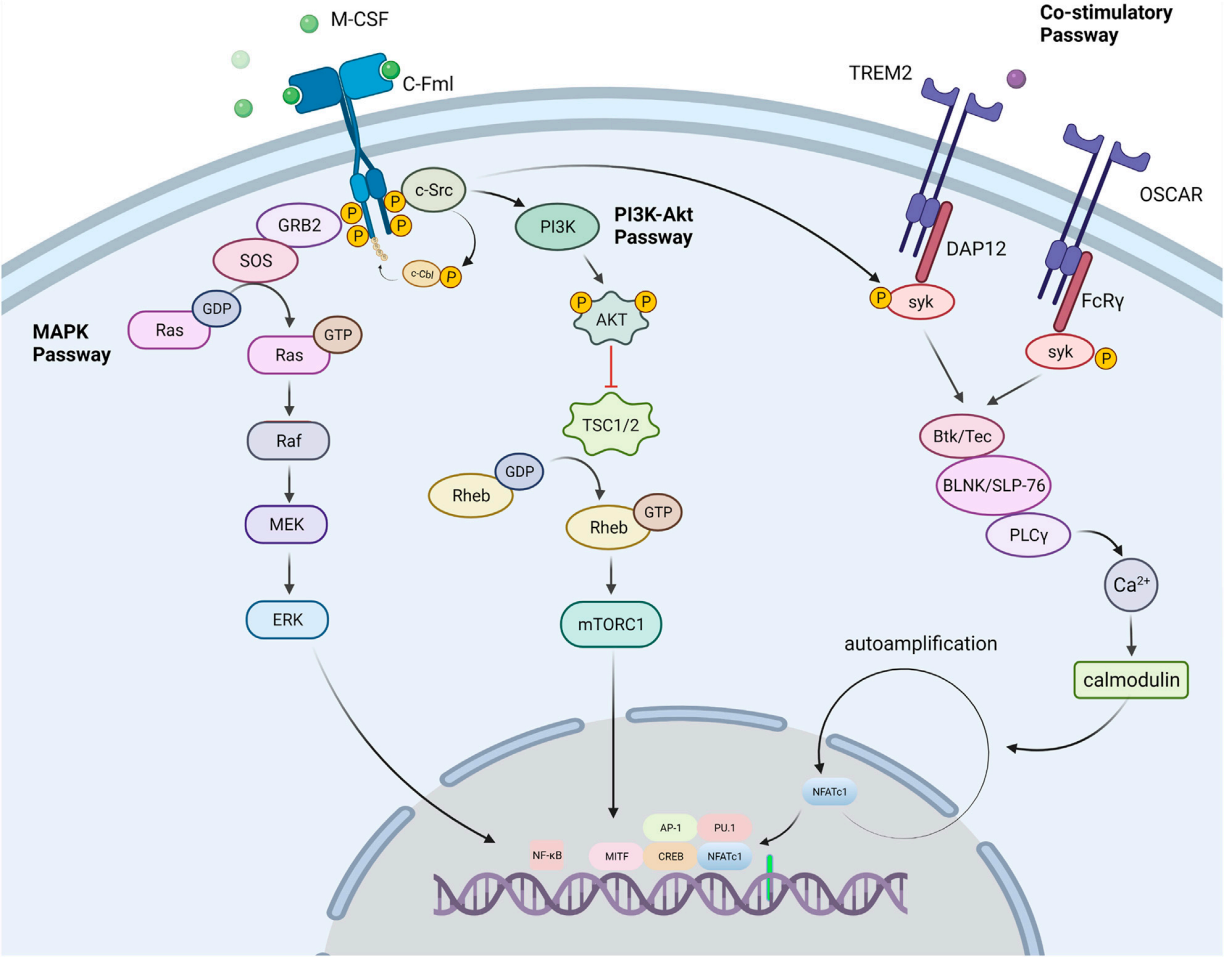
Signal passways in the process of osteoclastogenesis under the stimulation of M-CSF. PI3K-Akt pathway: M-CSF binds to its receptor c-Fms, induces partial phosphorylation of key sites in the c-Fms tail, and recruits the tyrosine kinase c-Src. The partially phosphorylated receptor binds to c-Src to form a c-Fms/c-Src Protein complex. Then the complex recruits E3 ubiquitin ligase (c-Cbl) and causes it to undergo tyrosine phosphorylation. As a response, the phosphorylated c-Cbl causes receptor ubiquitination, which further causes the receptor to be completely phosphorylated. Subsequently, the c-Fms/c-Src protein complex recruits phosphatidylinositol 3-kinase (PI3K) to trigger the PI3K-Akt pathway for OCPs activation and proliferation. MAPK pathway: After the receptor was stimulated, the receptor associated Grb2/Sos activates the MAPK signaling cascade, which conclusively leads to the phosphorylation and activation of the extracellular signal-regulated kinase (ERK) to promote the formation of osteoclasts. Co-Stimulatory pathway: M-CSF also promotes the co-stimulatory pathway of nuclear factor activated T cells 1 (NFATc1). M-CSF can activate the Fc receptor common gamma subunit (FcRγ) and dnax activation protein 12 (DAP12), which further activate the tyrosine kinase Syk. Syk forms a complex with Btk/Tec, BLNK/SLP76. The complex further activates PLCγ, initiates the PLC pathway, and dephosphorylates the transcription factor NFATc1 to promote its entry into the nucleus and induce the differentiation of precursor cells into osteoclasts.

M-CSF, also known as colony-stimulating factor 1 (CSF-1), is a dimeric glycoprotein linked by interchain disulfide bonds, mainly binding with colony-stimulating factor 1 receptor (CSF1R, also known as c-FMS), a receptor tyrosine kinase (Mun et al., 2020). The binding of CSF1R with M-CSF is crucial for the survival, function, proliferation, and differentiation of myeloid lineage cells, including osteoclasts, monocytes/macrophages, microglia, langerhans cells in the skin, and Paneth cells in the intestine (Mun et al., 2020). M-CSF promotes RANK expression on the OCPs membrane and enables RANK cells to initiate a response to RANKL (Ross and Teitelbaum, 2005). Some studies have certified the indispensable role of M-CSF, in which the *M-csf* gene is mutated and expresses non-functional M -CSF protein in mice (Wiktor-Jedrzejczak et al., 1990) As a result, these mice had fewer osteoclasts, leading to severe osteosclerosis. Several transcription factors, notably PU.1, and a heterodimeric complex of microphthalmia-associated transcription factor (MITF) and TFE3, cause myeloid progenitors to develop into OCPs. PU.1 and MITF stimulate the expression of the M-CSF receptor (c-Fms) (Iitsuka et al., 2012), and animals lacking either of these genes develop osteopetrosis. The tyrosine kinase receptor c-Fms is a transmembrane protein that contains numerous tyrosine residues in the cell. M-CSF binds to c-Fms and participates in osteoclast lineage differentiation by regulating a number of pathways including the PI3K-Akt, MAPK, and Co-Stimulatory pathways (Figure 2).

**FIGURE 3:**
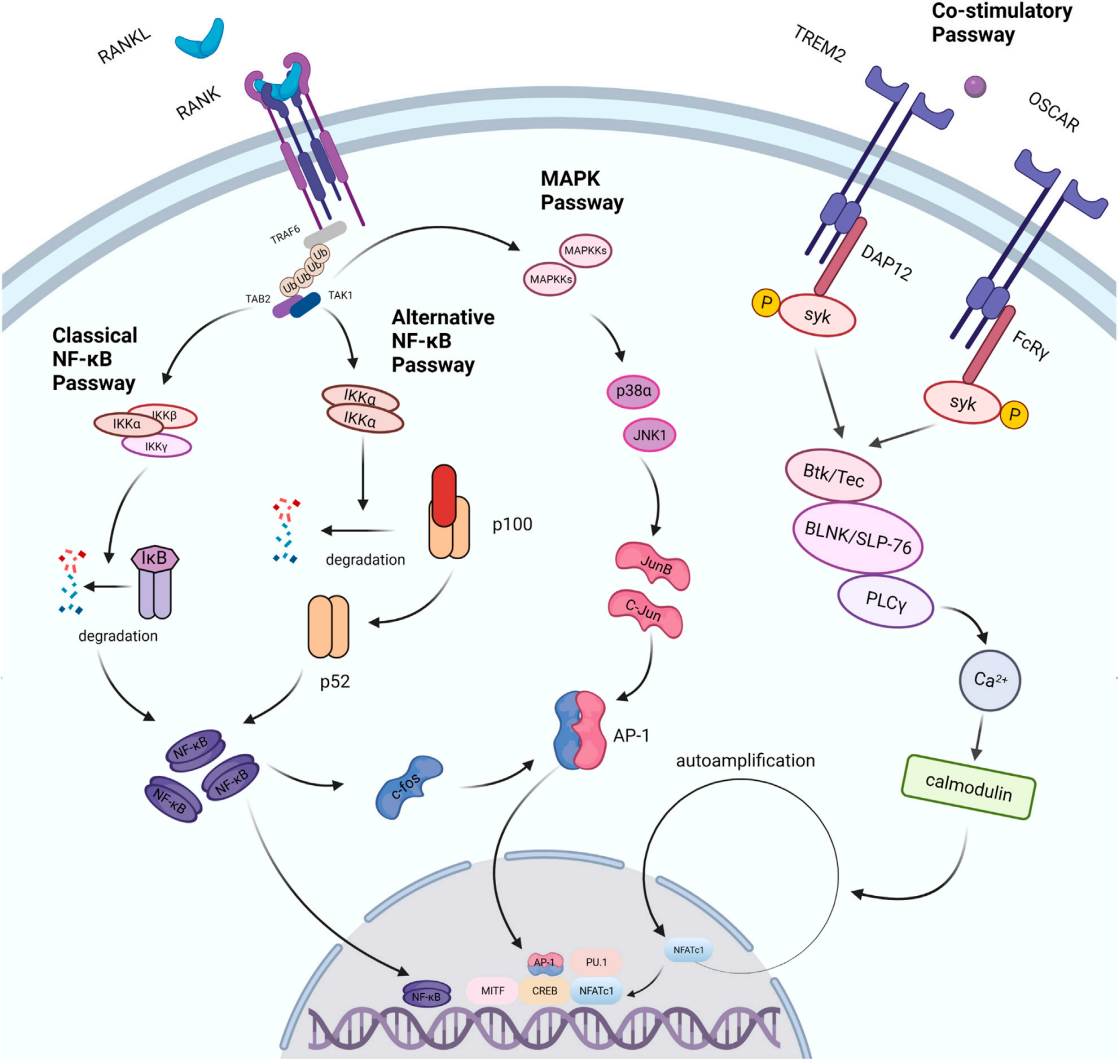
Signal passways in the process of osteoclastogenesis under the stimulation of RANKL. After RANKL binds to its receptor RANK, it transduces signals through the linker TRAF6, which recruits TAB2 and TAB1 to activate the MAPK pathway and the NF-κB pathway. NF-κB is one of the necessary transcription factors for osteoclast differentiation. The NF-κB pathway is divided into two types: the classic pathway and the alternative pathway. In the classical pathway, TRAFs activate the IKK complex (IKKα, IKKβ, IKKγ); under the action of the complex, the IκB molecule undergoes phosphorylation and ubiquitination, and then degrades to release NF- κB. In an alternative pathway, the homodimer of IKKα cleaves p100 to generate p52, which activates NF-κB. In the above two pathways, activated NF-κB dimer induces the expression of factors such as c-Fos and AP-1, and further drives the expression of NFATc1 to promote osteoclast differentiation. In addition, the expression of NFATc1 is also regulated by co-stimulatory pathway. FcRγ and DAP12 are important receptor molecules in the co-stimulatory pathway. They can activate the downstream tyrosine kinase Syk after binding to the corresponding ligand. Syk forms a complex with Btk/Tec, BLNK/SLP76, and then activates the PLC pathway to induce osteoclast differentiation. Studies have found that TRAF6 can directly activate c-Src and further trigger the PI3K/Akt pathway to promote osteoclast differentiation and proliferation.

RANKL is found in osteoblast series and immunocytes, but it’s more abundant in bone tissues (osteoblasts, osteocytes and mesenchymal) than in others (Nakashima et al., 2011). Purified osteocytes express RANKL more strongly than osteoblasts and support the formation of osteoclasts better, which indicates that osteocytes perform a vital function in the process of bone remodeling (Nakashima et al., 2011). RANKL is divided into free form and membrane-bound form, and the latter has higher activity (Lader et al., 2001). The differentiation process of osteoclasts is mainly regulated by RANKL. Loss of RANKL in mice results in severe osteopetrosis, while overexpression of soluble RANKL leads to severe osteoporosis (Kong et al., 1999; Mizuno et al., 2002). When monocytes expressing RANK, a member of the TNF receptor family, are exposed to RANKL, they merge to create multinucleated osteoclasts (Li Y.-X. et al., 2020). This procedure includes stimulation of several pathways including NF-κB pathway, MAPK pathway and co-stimulatory pathway (Figure 3). All of these pathways are involved in the production of NFATc1, the master osteoclastogenic transcription factor (Takayanagi et al., 2002).

Beyond RANKL, M-CSF, recent studies have also identified many other factors that influence osteoclast differentiation. Post-translational SUMO modifications are essential for regulating osteoclast formation. Expression of SUMO-specific protease SENP3 is down-regulated in OCPs during osteoclast differentiation. It was shown that mice with bone marrow-derived monocyte (BMDM) SENP3 deficiency exhibit more severe bone loss after oophorectomy due to osteoclast hyperactivation (Zhang Y. et al., 2020). According to a related study, long-term exposure to cadmium (Cd) results in reduced expression of P2X7, which inhibits the P2X7-PI3K-AKT signaling pathway, thereby further suppresses osteoclast differentiation (Ma et al., 2021). Selenoproteins containing selenium in the form of selenocysteine are critical for bone remodeling. Selenoprotein W ensures physiological bone remodeling by preventing hyperactivity of osteoclasts. Study identifies selenoprotein W (SELENOW) as a protein down-regulated through RANKL/RANK/tumor necrosis factor receptor-associated factor 6/p38 signaling by large-scale mRNA analysis of nuclear factor (NF)-κΒ ligand (RANKL)-induced osteoblast differentiation (Kim et al., 2021). RNA-sequencing analysis revealed that SELENOW regulates osteoclastogenic genes. *SELENOW* overexpression enhances osteoclastogenesis *in vitro via* nuclear translocation of NF-κB and nuclear factor of activated T-cells cytoplasmic 1 mediated by 14-3-3γ, whereas its deficiency suppresses osteoclast formation (Kim et al., 2021). Major vault protein (MVP) (also known as lung resistance-related protein, LRP), is the main component of cellular ribonucleoprotein particles called vaults. Vaults are highly conserved across species and most mammalian cell vaults are in the cytoplasm (Esfandiary et al., 2009). Research suggested that MVP negatively regulates osteoclast differentiation and bone resorption *via* inhibition of calcineurin-NFATc1 signaling and *Mvp−/−* and *Mvp* ^*f/f*^ *Lyz2-Cre* mice both exhibited osteoporosis-like phenotypes (Yuan L. et al., 2021). MVP-deficiency also enhanced calcineurin-NFATc1 signaling and promoted NFATc1 activity, which led to enhanced osteoclastogenesis and bone resorption (Yuan L. et al., 2021). Hh pathway, an evolutionarily conserved signaling pathway, plays critical roles in skeletal development and homeostasis, and is regarded as a promising anabolic pathway for treating osteoporosis and promoting bone regeneration. Researchers found that activated Hh signaling in macrophages can strongly inhibited RANKL-induced TRAP+ osteoclast production, F-actin ring formation, osteoclast-specific gene expression, and osteoclast activity *in vitro*. Mechanistic study revealed that activation of Hh signaling suppressed RANKL-induced activation of JNK pathway and downregulated protein levels of two key osteoclastic transcriptional factors, c-Fos and its downstream target NFATc1 (Zhang L. et al., 2020). In mouse/human bone specimens and mouse primary BMMs, miR-128 levels were found to be positively linked with higher *Nfatc1* levels and findings reveal a key mechanism of osteoclastogenesis mediated by the miR-128/SIRT1/NF-κB signaling axis (Shen et al., 2020). Exosomes, also known as extracellular vesicles, are naturally occurring, biocompatible, and bioacive nanoparticles ranging from 40 to 150 nm in diameter, which play important roles in bone homeostasis. Endothelial cell (EC)-secreted exosomes (EC-Exos) show more efficient bone targeting than osteoblast-derived exosomes or bone marrow mesenchymal stem cell-derived exosomes (Song et al., 2019). EC-Exos can inhibit osteoclast activity *in vitro* and inhibit osteoporosis in an ovariectomized mouse model (Song et al., 2019). Breast cancer exosomes contribute to osteoclast differentiation and promote bone metastasis of tumor cells (Yuan X. et al., 2021). CD137 can also promote bone metastasis of breast cancer by enhancing the migration and osteoclast differentiation of monocytes/macrophages (Jiang et al., 2019). In addition, high glucose, osteoblast-derived OPG, and notch signaling pathways were found to stunt osteoclast differentiation (Hu et al., 2019; Cawley et al., 2020; Ganguly et al., 2020). Irisin, melatonin, oleuropein and loureirin B also have regulatory effects on osteoclast differentiation (Ikegame et al., 2019; Liu et al., 2019; Estell et al., 2020; Rosillo et al., 2020). Two widely used drugs for the treatment of erectile dysfunction, tadalafil and vardenafil, trigger an increase in bone mass in mice through a combination of anabolic and anti-resorptive effects on bone, inhibiting the target enzyme, phosphodiesterase 5A (PDE5A) mechanistically (Kim S.-M. et al., 2020).

## 3 Osteoclast Apoptosis

### 3.1 Pathways of Apoptosis

Apoptosis in osteoclasts can be triggered by either the extrinsic (death receptor) or internal (mitochondria) pathways (Hengartner, 2000). Both pathways can activate caspase, which can induce apoptosis by cleaving specific substrates (Xing and Boyce, 2005). In addition to the above 2 classical apoptotic pathways, there are more new insights about osteoclast apoptotic pathways in recent years. Osteomorphs are a unique cell type from osteoclasts and macrophage progenitors (McDonald et al., 2021). Osteomorphs are produced by the fission of osteoclasts into smaller, more motile daughter cells that possess the ability to fuse to form new osteoclasts (McDonald et al., 2021). In contrast to osteoclasts that are attached to bone, osteomorphs are found in the bone marrow and blood (McDonald et al., 2021). Osteoclasts recycle *via* osteomorphs during RANKL-stimulated bone resorption was found recently (McDonald et al., 2021). Mature osteoclasts seperate into smaller osteomorphs allow them to persist and survive for extended periods of time until they are required again (McDonald et al., 2021). Accordingly, osteomorphs can re-fuse into multinucleated osteoblasts under the right conditions. This approach bypasses the traditional apoptotic pathway and provides a new insight into the destination of mature osteoclasts. Protective autophagy has long been thought to have an anti-apoptotic effect. Paradoxical effects of IL-17A on osteoclastogenesis reveal the relationship between autophagy and osteoclast apoptosis. It is well accepted that protective autophagy has an anti-apoptotic effect.Researchers revealed a phenomenon that OCPs’ apoptosis was differently modulated by various concentrations of IL-17A: apoptosis was promoted by high concentrations of IL-17A, whereas it was inhibited by low concentrations of IL-17A (Xue et al., 2019). However, apoptosis was decreased and autophagy was activated by over-expression of Beclin1 under high levels of IL-1 7A (Xue et al., 2019). Beclin1 silencing not only inhibited autophagy, but also up-regulated apoptosis in presence of low levels of IL-1 7A (Xue et al., 2019). Further research found autophagy enhances osteoclastogenesis by degrading TRAF3 (Xue et al., 2019). Therefore, Beclin1-autophagy-TRAF3 signaling pathway is regard as a novel pathway to regulate osteoclast apoptosis.

### 3.2 Pro-Apoptotic Factors

In addition to the traditional pro-apoptotic factors such as transforming growth factor B (TGF-B), estrogen, bisphosphonates, denosumab and raloxifene, many other factors have been identified in recent years. Long-term exposure to cadmium (Cd) results in reduced expression of P2X7, which inhibits the P2X7-PI3K-AKT signaling pathway, thereby further promotes osteoclast apoptosis (Ma et al., 2021). The newly characterized gene *Merlot*, which encodes a highly conserved yet uncharacterized protein in vertebrates, is an important regulator for termination of osteoclastogenesis *via* induction of apoptosis (Yamakawa et al., 2020). Krox20/EGR2 is a zinc-finger transcription factor associated with hindbrain development, neuromyelin formation and tumor suppression (Yamakawa et al., 2020). Recent studies have demonstrated that this factor promotes apoptosis in osteoblasts and has a significant sex dimorphism: the phenotype is restricted to females (Yamakawa et al., 2020). Tussilagone (Ryoo et al., 2020), triptolide (Wang et al., 2019) and W9 peptide (Kou et al., 2021) are also important factors in promoting osteoclast apoptosis.

### 3.3 Anti-Apoptotic Factors

For osteoclasts, classical anti-apoptotic factors include p65 protein, M-CSF, RANKL, TNF, IL-1, IL-3 and VEGF-A. In addition to the above, src inhibitors cause osteoclast apoptosis, but *Src−/−* mice osteoclast apoptosis does not increase because other factors compensate (Xing and Boyce, 2005). PTH and 1,25(OH)2-VitD3 can stimulate the expression of RANKL and reduce the expression of osteoprotegerin (OPG) to prevent osteoclast apoptosis, and their local concentration plays a decisive role in determining the survival and formation of osteoclasts (Gori et al., 2000). New study shows that myeloid-specific deletion of *Sirt6* led to decreased ERα protein level and apoptotic cell death in preosteoclasts, which indicates sirtuin 6 in preosteoclasts suppresses age- and estrogen deficiency-related bone loss by stabilizing estrogen receptor α (Moon et al., 2019).

### 3.4 Other Factors

While Fas is the major receptor for FasL, another member of the TNFRs, decoy receptor 3 (DcR3), also acts as a decoy receptor for FasL (Yang et al., 2004). DcR3 is a molecule that binds and blocks numerous TNF family proteins, not simply FasL, in a competitive manner. According to reports, DcR3 serves as a ligand, inducing macrophage transformation into osteoclasts. DcR3 also suppresses the production of osteoclasts triggered by RANKL *via* inhibiting the NF-kB and NFATc1 pathways (Cheng et al., 2013), thereby acting as a regulator of osteoclast apoptosis. osteoclasts could be induced to apoptosis by OPG produced by osteoblasts. On the other hand, OPG can bind to and block TNF-related apoptosis-inducing ligand (TRAIL), which causes osteoclast apoptosis. Through this mechanism, OPG appears to inhibit osteoclast apoptosis *in vitro* (Chamoux et al., 2008) but whether this mechanism affects *in vivo* needs further study. The effect of IL-17A on osteoblast apoptosis was dose-dependent, with high concentrations promoting apoptosis and low concentrations inhibiting it (Xue et al., 2019) Apoptotic bodies (ABs) traditionally considered as garbage bags that enclose residual components of dead cells are gaining increasing attentions due to their potential roles in intercellular communications (Ma et al., 2020). Both gene set and functional analysis indicated that osteoclast derived ABs are biologically similar with the parental cells suggesting their role in promoting bone defect healing (Ma et al., 2020). Currently, treatments targeting osteoclast-targeted apoptosis have been shown to alleviate a variety of diseases, including osteoarthritis (Deng et al., 2021).

## 4 Coupling Signals Between Osteoclast and Osteoblast

**FIGURE 4:**
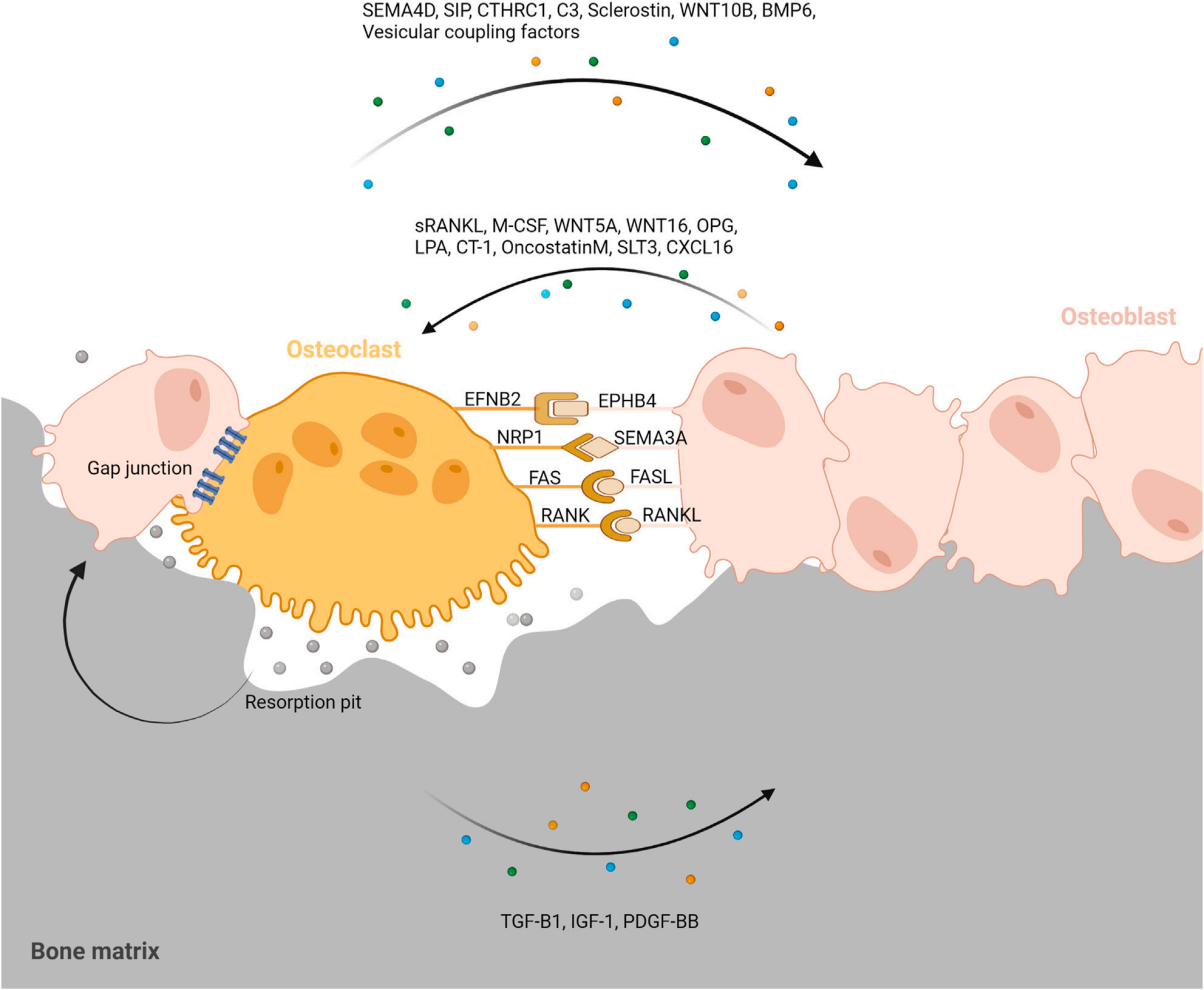
Coupling signals between osteoclast and osteoblast. To accomplish coordination between bone formation and resorption during bone remodeling, communication between osteoclasts and osteoblast lineage cells, as well as interactions with the resorption pit and others are essential. Coupling signals between osteoclast and osteoblast can be classified into five categories; The membrane-bound coupling signals are mainly composed of five coupling signals: gap junction, EFNB2/EPHB4, NRP1/SEMA3A, FAS/FASL, RANK/RANKL; Soluble coupling signals released from osteoblast mainly refer to the soluble factors acting on osteoclasts released by osteoblasts such as sRANKL, M-CSF, WNT5A, WNT16, OPG, LPA, CT-1, OncostatinM, SLT3, CXCL16; Soluble coupling signals released from osteoclast mainly refer to the soluble factors acting on osteoblasts released by osteoclasts such as SEMA4D, SIP, CTHRC1, C3, Sclerostin, WNT10B, BMP6, Vesicular coupling factors; Matrix-derived coupling signals are factors released by matrix which is mediated by osteoclast degradation during bone resorption such as TGF-B1, IGF-1, PDGF-BB; Vesicular coupling signals mainly refers to exosomes, microvesicles, and apoptotic bodies. These membrane-containing EVs can deliver proteins, lipids and mRNAs through exocytosis and endocytosis to promote coupling. Furthermore, the resorption pit, cellular canopy, and reversal phase could also play a role in the coupling between osteoclast and osteoblast.

Bone remodeling is critical for repairing and replacing damaged or aging bone. The temporal and spatial synchronization of bone resorption with bone formation is referred to as coupling during bone remodeling. The intricate interactions of osteoclasts and osteoblasts maintain bone homeostasis. Diverse processes mediate the coupling of bone formation to resorption during remodeling. To accomplish coordination between bone formation and resorption during bone remodeling, communication between osteoclasts and osteoblast lineage cells, as well as interactions with the canopy and the reversal phase, are essential. (Figure 4).

### 4.1 Membrane-Bound Coupling Signals

#### 4.1.1 Gap Junction

Bone lining cells (BLCs) cover inactive (nonremodeling) bone surfaces, particularly evident in the adult skeleton. BLC’s are thinly extended over bone surfaces, have flat or slightly ovoid nuclei, connect to other BLCs *via* gap junctions, and send cell processes into surface canaliculi. BLCs, an osteoblast subset, have been shown to connect with bone-bound osteoclasts (Everts et al., 2002). The interaction between those two cells is crucial for osteoclastogenesis to begin (Everts et al., 2002). A transmission electron micrograph also revealed that mature osteoclasts and osteoblasts have direct contact and that cell-cell contact occurs at the basic multicellular level (Matsuo and Irie, 2008).

#### 4.1.2 EFNB4(Ephrin B2)/EPHB4

Osteoblasts and osteoclasts interact through cell-to-cell direct contact during bone remodeling (Everts et al., 2002). Ephrin signaling can mediate this interaction (Zhao et al., 2006; Takyar et al., 2013; Tonna et al., 2016). Ephrin B (B1∼B3), the cell-surface molecules, interacts with EPHB (B1∼B6), which is their cognate receptors. The Ephrin B protein family is made up of transmembrane proteins that have cytoplasmic domains, mediating bidirectional signal transduction by interacting with EPHB-expressing cells. Ephrin B2 (EFNB2) located on the osteoclast membrane, binds to the EPHB4 receptor on the surface of osteoblasts. It is believed that EPHB4-mediated activation of EFNB2 initiates backward signaling (from osteoblast to osteoclast), which reduces osteoclast development by inhibiting the osteoclastogenic c-Fos/NFATc1 signaling pathway. Forward signaling (from osteoclast to osteoblast) is enhanced by EFNB2-induced activation of EPHB4, which promotes osteoblast development while inhibiting apoptosis (Tonna et al., 2014). Correspondingly, in a transgenic mouse model, excessive expression of EPHB4 in osteoblasts also enhances bone density (Zhao et al., 2006).

#### 4.1.3 FASL/FAS

FASL/FAS pathway is one of the two primary pathways on osteoclast apoptosis, and estrogen-induced osteoclast apoptosis is mediated by this mechanism. Estrogen-induced upregulation of FASL in osteoblasts causes pre-osteoclast apoptosis, indicating that the survival of osteoclasts is aided by a paracrine signal produced by osteoblasts. Furthermore, conditional deletion of *Fasl* in osteoblasts increases the number and function of osteoclasts, leading to a reduction in bone density (Wang et al., 2015).

#### 4.1.4 Semaphorin 3A (SEMA3A)/NRP1

Axonal growth cone guidance molecules are known as semaphorins, which are found in a wide range of tissues, such as the brain and skeleton (Negishi-Koga et al., 2011). Although SEMA3A is involved in the formation of central nervous system components, several studies suggest that it is also involved in bone modeling and remodeling (Behar et al., 1996; Fukuda et al., 2013). *In vivo*, sensory neuron-derived SEMA3A is needed for normal bone growth, according to certain studies (Fukuda et al., 2013). Additional research has identified that SEMA3A secreted by osteoblast inhibits osteoclast differentiation and promotes bone formation (Hayashi et al., 2012). They also discovered that when NRP1 expression was suppressed by short shRNA, SAMA3A’s inhibitory effect on osteoclast development was eliminated, demonstrating that SAMA3A suppresses osteoclastogenesis by binding to NRP1. By the WNT/B-catenin pathway, its binding to NRP1 reduces RANKL-induced osteoclast differentiation and promotes osteoblast differentiation (Hayashi et al., 2012).

#### 4.1.5 RANKL/RANK

Outside-in or reverse signaling within osteoblasts by RANKL is another membrane-bound coupling activity that has just been discovered. When it was observed that a RANKL-binding agent that inhibited osteoclast production also improved bone formation *in vivo* and encouraged osteoblast differentiation *in vitro*, this mechanism was discovered (Adams et al., 2006). Knocking down *Rankl* in the target osteoblastic cells prevented the latter impact, implying that RANKL signaling inside osteoblast precursors was to blame (Adams et al., 2006). This was validated in an inflammatory arthritic animal model, where the RANKL-binding agent both inhibited bone resorption and encouraged bone formation (Kato et al., 2015). Understanding the mechanisms is especially essential now that anti-RANKL treatment for osteoporosis is becoming more widely used. Recent research has found that, while RANKL reverse signaling is a membrane-bound activity, it is mediated *via* the release of extracellular vesicles (EVs) from the osteoclast rather by cell–cell interaction.

### 4.2 Soluble Coupling Signals Released From Osteoblast

#### 4.2.1 sRANKL

Soluble RANKL (sRANKL), which is secreted by osteoblasts, could connect with its receptor RANK, which is present on OCPs, activating downstream signaling pathways involved in cell development and maturation. Mice lacking RANK or sRANKL have identical phenotypes, suggesting that the sRANKL/RANK signaling axis is critical for bone remodeling (Kong et al., 1999; Li et al., 2000). In mice, the omission of *sRANKL* leads to serious osteopetrosis because of lack of osteoclasts, whereas overproduction of sRANKL causes significant osteoporosis (Kong et al., 1999; Mizuno et al., 2002). Therefore, for osteoporosis and related skeletal disorders, inhibiting sRANKL signaling is a potential therapeutic objective.

#### 4.2.2 M-CSF

Osteoblasts can secrete M-CSF, which binds to its receptor c-Fms expressed on macrophages and osteoclasts. At an early age, mice with a thymidine insertion in the *M-csf* gene, which causes M-CSF deficit, show a decrease in macrophages and osteoclasts (Kim J.-M. et al., 2020). Nevertheless, these traits vanish with age. In these mice, application with recombinant M-CSF or generation of soluble M-CSF from osteoblasts increases the number of osteoclasts and alters the phenotypes of osteopetrosis, indicating that M-CSF is needed for the generation of osteoclasts in youthful mice, but does not rule out the possibility of compensating systems that are not relying on M-CSF (Kim J.-M. et al., 2020).

#### 4.2.3 WNT5A

The WNT system regulates osteoblastogenesis and osteoclastogenesis *via* both B-catenin-dependent (canonical) and -independent (noncanonical) mechanisms, which are both important for maintaining bone balance. WNT5A, a noncanonical WNT ligand, is highly produced in osteoblasts and combines with the tyrosine kinase-like orphan receptor 2 (ROR2) on osteoclast membranes (Oishi et al., 2003). WNT5A enhances RANKL-induced osteoclastogenesis by activating the Jun–N-terminal kinase (JNK) MAPK cascade, which upregulates RANK expression in osteoclasts. The conversion of bone marrow-derived monocytes (BMM) into mature osteoclasts was hindered in mice with heterozygous ablation of *Wnt5a* or *Ror2*. Mice with osteoblast-targeted knockout of *Wnt5a* or osteoclast-target knockout of *Ror2* showed similar abnormalities in osteoclastogenesis (Movérare-Skrtic et al., 2014).

#### 4.2.4 WNT16

In humans, the *WNT16* gene is tightly correlated to cortical bone thickness, BMD, and fracture risk. WNT16 is mainly derived from osteoblasts, and it is almost not expressed in osteoclasts (Movérare-Skrtic et al., 2014). WNT16 has a direct and indirect inhibitory effect on osteoclastogenesis. WNT16-induced JUN phosphorylation enhances OPG expression in osteoblasts, allowing for both direct and indirect inhibition of osteoclastogenesis *via* the noncanonical JNK MAPK cascade (Kim J.-M. et al., 2020). Researches have shown that *Wnt16* deletion causes a specific loss in cortical bone density and an increase in cortical porousness, as well as spontaneous fractures with no trabecular bone changes.

#### 4.2.5 OPG

OPG, also known as osteoclastogenesis inhibitory factor (OCIF), is a secreted glycoprotein produced by cells in the lungs, liver, and spleen (Simonet et al., 1997; Yasuda et al., 1998; Li Y. et al., 2007). B cells have been found to be the predominant source of OPG in mouse bone marrow, accounting for 64% of all bone marrow OPG production (Li X. et al., 2007). OPG, as a decoy receptor, could bind to RANKL and block its binding to RANK. As a result, the primary signaling pathway of osteoclast differentiation and activation was shut down. Osteopetrosis is observed in mice over-expressing OPG due to the absence of osteoclasts (Simonet et al., 1997). *Tnfrsf11b* (OPG) knockout mice have osteoporosis as a result of uncontrolled osteoclasts (Bucay et al., 1998).

#### 4.2.6 LPA

LPA is a bioactive phospholipid produced by a variety of cells, such as osteoblasts and activated platelets. Osteoblast-derived LPA could adjust osteoclast formation and apoptosis (Panupinthu et al., 2008). In osteoclasts, the LPA receptors LPA1, LPA2, LPA4, and LPA5 are expressed at differing proportions (Del Fattore et al., 2008). The binding of LPA to the receptor has the potential to control calcium signaling and cause NFATc1 nuclear accumulation in osteoclasts (Lapierre et al., 2010) which enhances OCPs fusion and promotes osteoclastogenesis (David et al., 2010; McMichael et al., 2010). LPA was also found to suppress osteoclast apoptosis and induce morphological changes in mature osteoclasts (Lapierre et al., 2010).

### 4.3 Soluble Coupling Signals Released From Osteoclast

#### 4.3.1 Semaphorin 4D (SEMA4D)

Sema4D is a molecule involved in axon guidance that is significantly expressed in osteoclasts (Negishi-Koga et al., 2011). SEMA4D, which is produced by osteoclasts, binds to Plexin-B1 on the membrane of osteoblasts and prevents their differentiation (Negishi-Koga et al., 2011). Mechanistically, SEMA4D binding to PLXNB1 triggers the small GTPase RHOA, suppressing osteoblast differentiation by inhibiting insulin-like growth factor-1 (IGF-1) signaling (Kim J.-M. et al., 2020). Sema4D may also influence the localisation of osteoblasts to a specific location by inducing osteoblast migration *via* RhoA activation (Zhang et al., 2015). *Sema4d*-deficient mice had significantly higher bone density, trabecular thickness, and bone strength than wild-type mice, according to research. Anti-SEMA4D antibody treatment prevents bone loss and encourages bone development in a mouse model of postmenopausal osteoporosis without influencing osteoclast-mediated bone resorption, indicating that SEMA4D may be a possible pharmacological object for osteoporosis and other decreased bone density diseases (Pederson et al., 2008).

#### 4.3.2 Cardiotrophin-1 (CT-1)

Although the number of osteoclasts in mice with total *Ct-1* deletion is high, their resorptive efficiency and bone formation are low, suggesting that coupling factor production is reduced (Walker et al., 2008). CT-1 was found in osteoclasts *in situ* and has been demonstrated to induce osteoblast development *in vitro* and bone growth *in vivo* (Walker et al., 2008). Despite the fact that the biological source of CT-1 within bone appears to be limited to osteoclasts, CT-1 stimulates bone formation *via* several pathways. These include actions on early precursors to promote osteoblast development at the expense of adipogenesis (Walker et al., 2008) and actions on osteocytes to inhibit sclerostin synthesis (Walker et al., 2010). CT-1 also promotes the expression of RANKL in the osteoblast lineage, which encourages the production of osteoclasts (Richards et al., 2000). As a result, CT-1 has a variety of effects, including coupling factor activity.

#### 4.3.3 Sphingosine 1 Phosphate (S1P)

Sphingosine is phosphorylated by sphingosine kinase (SPHK) to produce sphingosine 1 phosphate (S1P), which stimulates osteoblastogenesis (Pederson et al., 2008; Ishii et al., 2009). RANKL stimulation on OCPs can increase S1P production and S1P attaches to the S1P receptor found on osteoblasts, causing them to migrate and survive more. The expression of RANKL is then upregulated by S1P-activated osteoblasts, resulting in promoting osteoclast formation (Ryu et al., 2006). Additional researches have revealed that S1P is involved in the control of osteoclastogenesis as well as communication between osteoclasts and osteoblasts or T cells (Ryu et al., 2006).

#### 4.3.4 Collagen Triple Helix Repeat Containing 1 (CTHRC1)

The soluble molecule CTHRC1 is generated by mature osteoclasts, which stimulates osteoblast differentiation in stromal cells (Takeshita et al., 2013). Although the CTHRC1 receptor in osteoblast has yet to be detected, recombinant CTHRC1 can promote bone formation by causing stromal cell recruitment and osteoblastic differentiation. Correspondingly, *Cthrc1* deletion in osteoclasts causes reduced bone density and impaired bone production. As mature osteoclasts come into touch with hydroxyapatite and calcium, *Cthrc1* expression increases, according to new research (Takeshita et al., 2013).

#### 4.3.5 Complement Component C (C3)

During osteoclastogenesis, C3 expression is increased, and C3 generated from osteoclasts is degraded to C3a, resulting in the induction of osteoblastogenesis (Matsuoka et al., 2014). C3a binds to its receptor expressed on stromal cell lines and primary calvarial osteoblasts. A C3a receptor (C3AR) antagonist reduces the osteogenic activity of conditioned medium derived from osteoclast, whereas a C3AR agonist stimulates osteoblast differentiation. The expression of C3 in bone is significantly increased in ovariectomies (OVX)-induced osteoporosis, and a C3aR antagonist prevents bone formation in OVX mice, which suggests C3a might regulate the pairing of bone resorption to production in a high turnover model (Matsuoka et al., 2014).

#### 4.3.6 Other Factors

WNT/B-catenin signal is one of the important signals regulating cell proliferation and differentiation, and WNT10B is a ligand of WNT/B-catenin signal (Chen et al., 2017). WNT10B induces osteoblast maturation and causes human bone marrow stromal cells to become more mineralized through activating the canonical WNT signaling pathway (Bennett et al., 2005; Pederson et al., 2008). *Wnt10b*-deficient mice have lower trabecular bone mass and osteocalcin levels in their blood (Pederson et al., 2008). Sclerostin, which is encoded by the *SOST* gene, is an anti-anabolic protein that inhibits bone growth (Balemans et al., 2001; Winkler et al., 2003). A study found that sclerostin is generated in osteoclasts of elderly mice (Ota et al., 2013). Mechanistically, Sclerostin inhibited the function of BMP6 and BMP7 in mouse MC3T3-E1 cells by binding to them with high affinity (Kusu et al., 2003). Human mesenchymal stromal cells migrate and mineralize in the presence of BMP6 in osteoclast-conditioned media, indicating it may play a role in coupling osteoclasts and osteoblasts in addition to Vesicular RANK (Pederson et al., 2008). OncostatinM, SLT3, CXCL16 also factors secreted by osteoclasts (Sims and Martin, 2020).

### 4.4 Matrix-Derived Coupling Signals

#### 4.4.1 TGF-B1

One of the most prevalent proteins in the bone matrix, TGF-B1, modulates osteoblasts and osteoclasts, assisting to remodel the bone (Kim J.-M. et al., 2020). TGF-B1 is non-covalently binding to the bone matrix protein latency-associated protein (LAP), which maintains it dormant by hiding the TGF-B1 receptor-binding regions (Dallas et al., 1994; Sengle et al., 2011). As a result, TGF-B1 resides inactive in the bone matrix until it is secreted during osteoclastic bone resorption (Tang et al., 2009). The bone mesenchymal lineage cells are then recruited by functional TGF-B1, leading them to move to resorptive sites and develop into bone-forming osteoblasts.

#### 4.4.2 IGF-1

The insulin-like growth factor is an important regulator of osteogenesis (Bautista et al., 1990). Among them, insulin-like growth factor 1 (IGF-1) is the most plentiful growth factor in the bone matrix, capable of maintaining bone mass in adults. Mechanically, the acidic environment during bone resorption activates IGF-1, and activated IGF-1 stimulates the osteoblast differentiation of recruited mesenchymal stem cells (MSC) by activating the mammalian target of rapamycin (mTOR), thus keeping apt bone microstructure and quality (Xian et al., 2012). Mice lacking the IGF-1 receptor (IGF-1R) in preosteoblast cells have lower bone mass and mineral deposition rates than wild-type mice, according to relevant research.

#### 4.4.3 PDGF-BB

Another growth factor that could play a role in the coupling mechanism is homodimeric platelet-derived growth factor, which is made up of two B units (PDGF-BB). Both osteoblasts and osteoclasts produce PDGF-BB, which is also released from the matrix (Canalis, 2021). The ability of PDGF-BB to induce blood vessel formationmay also provide progenitor cells for later differentiation into osteoblasts and bone formation (Xie et al., 2014).

### 4.5 Vesicular Coupling Signals

Cells express a variety of membrane-containing EVs, such as exosomes, microvesicles, and apoptotic bodies (van der Pol et al., 2012). EVs are discharged from the cell *via* exocytosis and can connect with target cell surface receptors as well as transfer intracellular components such as proteins, lipids, messenger RNAs (mRNAs), and microRNAs to the target cell’s cytosol *via* endocytosis. The target cell could be proximate, or the EVs could be delivered to a more distant place, including possible circulation. EV transport of membrane-bound RANK and microRNAs may represent additional coupling mechanisms within the BMU. EVs have been reported to be released by osteoclasts. (Huynh et al., 2016): Exosomes were discovered in cell cultures containing both osteoclast progenitors and developed osteoclasts using electron microscopy. A small percentage of these EVs were enriched for RANK on the membrane and suppressed osteoclast formation *in vitro*. Recently, it was discovered that such vesicles containing RANK produced by mature osteoclasts increased bone production by triggering RANKL reverse signaling to activate Runx2 (Ikebuchi et al., 2018). This would imply that EVs enriched for RANK on their cellular membrane could stimulate reverse RANKL signaling in the early osteoblast lineage.

## 5 Other Mechanisms to Promote Coupling

### 5.1 The Effect of the Resorption Pit

The ways by which osteoclasts transmit coupling signals are not restricted to matrix release, signaling molecule secretion, or microvesicle release. Osteoclasts also transmit signal by leaving a resorptive pit once resorption is complete. Once attracted to the resorbed bone surface, osteoblast lineage cells may detect changes in topography. When rat calvarial cells were cultivated on bone slices having crevices created by osteoclasts or mechanically excavated grooves, the cells produced bone preferentially in those flaws, filling them to a flat surface (Gray et al., 1996). This shows that, while chemicals may be necessary to bring cells to the surface, it is the architecture of the bone that directs them. Osteoclasts exert remote control over osteoblast activity by defining the size and shape of the resorptive pit to be filled. Once this process is begun, the participating cells must also detect the spatial limitations and communicate with one another *via* chemical communication when the space has been filled. This may entail gap junctions or communication processes between osteoblasts that are dependent on cell contact (Tonna and Sims, 2014). Because these *in vitro* studies used bone lacking osteocytes (Gray et al., 1996), the osteocytes are not necessary for osteoblasts to respond to topographic clues, at least *in vitro*. They may, however, have a distinct role during the *in vivo* filling of pits left by osteoclasts. Osteocytes detect and respond to mechanical strain *via* their fluid-filled lacunocanalicular network of communication channels. This highly complex communication system (Buenzli and Sims, 2015) might provide an additional coupling mechanism.

### 5.2 A Cellular Canopy as a Mechanism to Promote Coupling

Researchers first postulated the existence of a cellular canopy that arises during the onset of remodeling and extends over the active BMU. Hauge took many years to identify the bone remodeling compartment (BRC) in human biopsies (Hauge et al., 2001). It was proposed that lining cells detached from the bone surface at the beginning of the remodeling cycle and established a distinct compartment that migrated with the osteoclast throughout the remodeling cycle.

BRCs were postulated to involve sinusoidal endothelial cells and to function as a component of the circulatory system. The vasculature’s link to the BRC provided a pathway for osteoclast precursors or even partially differentiated inactive osteoclast precursors (Mizoguchi et al., 2009). Capillaries linked with the canopy also enable for the entry of other cells, such as mesenchymal precursors (Eghbali-Fatourechi et al., 2007), immunological, and endothelial cells.

The canopy, it has been proposed, not only creates a separate BRC but is also essential for the reversal phase to be completed. This is based on the finding of incomplete canopies at locations of reversal phase halt in biopsies from osteoporotic patients; these are unusual regions of uncoupling where bone formation is not detected after bone resorption (Andersen et al., 2014; Jensen et al., 2015). It’s possible that the canopy keeps local coupling factor concentrations high enough to facilitate precursor recruitment or drive osteoblast differentiation and bone formation. Osteoblast lineage cells, osteoclasts, endothelial cells, vascular cells, and immune cells may exchange factors and impact precursors given by the related vasculature in this BRC (Kristensen et al., 2013); neuronal cells may also come into close contact with the canopy at active remodeling locations, according to recent research (Sayilekshmy et al., 2019). It’s also been suggested that the osteoblast lineage cells that make up the canopy contain target cells for coupling activity (Delaisse, 2014), which could be a way by which membrane-bound osteoclast-derived substances come into touch with osteoblast precursors or perhaps the canopy cells themselves. In this situation, the signal to bone lining cells in touch with the osteoclast to raise the canopy would contribute to the coupling process.

### 5.3 The Reversal Phase

The reversal phase is the stage between bone formation and bone resorption. Bone lining cells were discovered at the bottom of resorption pits near the end of resorption, where they clear demineralized collagen to pave the pits for the involvement of osteoblasts to produce bone (Villanueva et al., 1986; Everts et al., 2002). This discovery opened the possibility that they could be stimulated to become matrix-producing osteoblasts, as seen on bone surfaces in PTH-treated animals (Kim et al., 2012). When *in situ* hybridization and immunohistochemistry were utilized in BMUs from human trabecular and Haversian (cortical) bone, the possibility was confirmed (Abdelgawad et al., 2016; Lassen et al., 2017). From lining cells near osteoclasts to plump, active osteoblasts near bone-forming surfaces, there was a consistent transition in marker expression and cellular morphology (Abdelgawad et al., 2016). This points to a reversal phase in which osteoblast differentiation proceeds until a sufficient mass of mature osteoblasts is achieved, after which matrix formation takes place (Abdelgawad et al., 2016).

On the reversal phase surface, smaller than typical osteoclasts were found sparsely scattered amid the osteoblast lineage cells, which was a novel finding. Their numbers fell as they got further away from the resorption pit, showing that they were dwindling in numbers following resorption. These osteoclasts are expected to signal neighbouring osteoblast lineage cells through any of the following mechanisms: matrix-derived protein release, protein secretion, EV release, and, most crucially, membrane-bound protein expression, given their likely interaction with osteoblast lineage cells. Because of the dispersed nature of osteoclasts on the reverse surface, membrane-associated signalling may be a small factor, but they may produce activities that aid in the osteoblast differentiation that appears to be occurring there (Lassen et al., 2017). The discovery of the reversal phase complicates the simple model of bone remodeling and the role of the reversal phase still needs to be further explored.

## 6 Behavior Changes of Osteoclast

The behavior of osteoclasts is regulated by several factors. Recently, the incidence of senile osteoporosis and postmenopausal osteoporosis has been increasing year by year, suggesting that aging and gender may be important factors contributing to changes in osteoclast behavior. In the next sections, we will discuss aging-induced or sex-associated behavior changes of osteoclast separately.

### 6.1 Aging-Induced Behavior Changes of Osteoclast

Cellular senescence, defined as the exit from the cell cycle accompanied with the acquisition of the senescence related secretory phenotype, plays a crucial role in health and illness, as well as embryonic tissue remodeling (Gorissen et al., 2018). An excess of bone resorption is a characteristic of bone aging. The osteoclast is a component of bone tissue that is responsible for bone resorption. In human marrow cells, there are age-related variations in the expression of osteoclast differentiation factors and receptors such RANKL/RANK/OPG and M-CSF (Chung et al., 2014). Sclerostin is produced by osteoclasts in aged mice, which may contribute to bone formation deficiency in the elderly. (Chung et al., 2014). Osteoclasts require relatively low levels of ROS for differentiation and activity. Caspase-2 deficiency increases oxidant resistance, as seen by decreased oxidative stress-induced osteoclast apoptosis. Caspase-2 regulates bone homeostasis by prompting oxidatively damaged osteoclasts to apoptose (Sharma et al., 2014). Unlike the decline of osteoblast function, osteoclast activity is maintained and even reactivated during senescence because of reactive oxygen species (ROS) production. ROS increases with advancing age, in part because the ability of cells to scavenge ROS decreases progressively with lifespan (Sharpless and DePinho, 2007). The behavior and development of osteoclasts are regulated by ROS, which has been known for many years (Garrett et al., 1990; Bax et al., 1992; Steinbeck et al., 1994; Darden et al., 1996; Lee et al., 2005). Exogenous ROS exposure especially H_2_O_2_ causes the RANK signaling cascade to become activated, resulting in the development of osteoclasts (Bax et al., 1992), whereas RANKL initiation causes the synthesis of endogenous ROS, which subsequently function as a second messenger to cause conversion into osteoclast (Callaway and Jiang, 2015). RANKL could enhance the level of intracellular ROS throughout osteoclastogenesis through initiating signaling pathways that include tumor necrosis factor receptor (TNFR)-associated factor 6 (TRAF6) and Nox1 (Lee et al., 2005). There is increasing evidence that the activities of important osteoclast transcription factors such as NF-kB and NFATc1 may also be influenced by ROS (Liu et al., 2019). Furthermore, the production of ROS by RANKL-stimulated osteoclasts has been shown to suppress the synthesis of antioxidant proteins like catalase (CAT) and superoxide dismutase (SOD) (Park et al., 2014). As a result, ROS could be regarded as a crucial signaling messenger during osteoclastgenesis.

EV-mediated signaling also play a role in osteoclast aging. A recent study has shown that EV generated from osteoclasts can transfer miR-214-3p to osteoblasts and suppress osteogenesis *in vitro* and *in vivo* (Li D. et al., 2016). Elevated expression of miR-214-3p in human bone biopsies was linked to increased serum exosomal miR-214-3p, and serum miR-21-3p levels were higher in elderly patients with fractures compared to age-matched controls without fractures, according to their findings. They also discovered that females’ serum levels of miR-214-3p increased with age, regardless of fracture status. The main source of serum exosomal miR-214-3p was osteoclasts, not osteoblasts. In an elderly mouse model of ovariectomy-induced bone loss, elevated osteoclastic and serum miR-214-3p were also linked to lower bone formation rates. Inhibition of miR-214-3p with an osteoclast-targeted antagomir enhanced bone production in mice, while targeting miR-214-3p overexpression to osteoclasts decreased bone formation. Based on this, there is a clear need for future studies to further delineate the role of exosomes in osteoclast in aging as well as studies aimed at understanding the mechanisms governing their release and uptake by cells (Qin and Dallas, 2019).

Recent findings identify a novel Cx43/miR21/HMGB1/RANKL pathway involved in osteoclast formation/recruitment (Davis et al., 2017). This pathway becomes impaired with age, resulting in increased osteoclastogenesis.

Sirtuin-3 (Sirt3) is an important metabolic regulatory enzyme that activates mTOR signaling and thus promotes osteoclastogenesis (Ho et al., 2017). Notably, Sirt3 expression is increased in osteoclasts during aging, suggesting that Sirt3 promotes age-related bone loss (Ho et al., 2017). Reduced TLR4 expression on the surface of osteoclast precursors and expansion of the osteoclast precursor pool also contribute to the aging-induced increase in osteoclast activity (Cao et al., 2005; Akkaoui et al., 2021). Aging of bone increased CATK-mediated osteoclastic resorption by 27% (Panwar et al., 2015). Macrophage/osteoclast-specific deletion of *Smo* in mice was found to attenuate the aging phenotype characterized by trabecular low bone mass, suggesting that blockage of the Hh-signaling pathway in the osteoclast lineage plays a protective role against age-related bone loss (Kohara et al., 2020). Hypoxia negatively affects osteoclast senescence and delays osteoclast formation (Gorissen et al., 2018). In terms of cells, elimination of OCPs had no significant effect on age-related bone loss (Kim et al., 2019) whereas macrophages promoted osteoclast production during aging (Kanagasabapathy et al., 2020). Furthermore, doxercalferol (DOX) (Li J. et al., 2020), Cnr1 and Cnr2 receptors (Sophocleous et al., 2017), cadherin-13 (Yang et al., 2020), GD3 Synthase (Yo et al., 2019), and Kynurenine (Refaey et al., 2017) all have a non-negligible role in aging osteoblasts.

### 6.2 Sex-Associated Behavior Changes of Osteoclast

#### 6.2.1 Estrogens

Osteoclasts express estrogen receptor alpha (Erα) (Oursler et al., 1991), and targeting Erα deletion in myeloid cells, which comprise the osteoclast progenitor, resulted in an increased osteoclast quantity and reduced trabecular bone mass phenotype in mice (Martin-Millan et al., 2010). The absence of Erα in myeloid cells resulted in a bone morphology similar to that seen in ovariectomized animals. Additionally, unlike wild type mice, ovariectomizing these mice did not result in a further loss in trabecular bone mass or an increase in trabecular osteoclast quantity. These findings suggest that expression of Erα in myeloid cells, including osteoclasts, is involved in the loss of trabecular bone mass in mice. Surprisingly, these researchers discovered that animals with Erα deletion in myeloid cells lost cortical bone mass after ovariectomy (Martin-Millan et al., 2010). As a result, it seems that Erα expression in osteoclasts does not play a role in the loss of cortical bone mass in mice. The researchers also proved that non-nuclear Erα binding in myeloid cells was important for estrogen’s beneficial effects on trabecular bone using a sequence of genetic substitutions and particular nuclear Erα ligands.

Estrogens increase apoptosis and prevent resorption in osteoclasts (Kameda et al., 1997) *via* Fas ligand (FasL), Fas receptor (Nakamura et al., 2007; Krum et al., 2008; Kovacic et al., 2010), and TGF-B signaling (Hughes et al., 1996; Robinson et al., 1996). In mice with estrogen deficiency due to ovariectomy, loss of ERα in mature osteoclasts resulted in an increase in FasL expression (Nakamura et al., 2007). In mice with estrogen deficiency due to ovariectomy, loss of ERα in mature osteoclasts resulted in an increase in FasL expression (Martin-Millan et al., 2010). The cause of this disparity is unknown. The effects of estrogen on osteoclast mitochondrial oxidative phosphorylation have also been addressed (Kim H.-N. et al., 2020). Female osteoclasts with deleted Era, but not males, showed trabecular bone loss, which was identical to the osteoporotic bone phenotype seen in postmenopausal women (Nakamura et al., 2007; Martin-Millan et al., 2010). Furthermore, estrogen increased apoptosis and raised FasL expression in WT, but not Erα deficient mice’s trabecular bone osteoclasts (Nakamura et al., 2007). FasL synthesis by osteoblasts in response to estrogen has also been shown to act as a paracrine regulator of osteoclast apoptosis (Krum et al., 2008). Importantly, the latter authors were unable to show that estrogen withdrawal caused overexpression of FasL in osteoclasts. As a result, this point is still debatable.

The ability of ovariectomy and subsequent estrogen withdrawal to lengthen the life span of osteoclasts was also discovered to be prevented by antibody suppression of TGF-B (Hughes et al., 1996). Interaction of Erα with the adaptor protein breast cancer anti-estrogen resistance protein 1 (BCAR1) (Robinson et al., 2009) and production of the tyrosine kinase Lyn in osteoclasts appear to be required for these effects (Gavali et al., 2019). Osteoblasts, osteocytes, and osteoclasts also express ERβ (Crusodé de Souza et al., 2009). Its role in these cells, however, is less well understood. Estrogen also has effects on osteoclastic bone resorption and trabecular bone mass, but not cortical bone mass, which are mediated through changes in the gut wall’s permeability to bacterial metabolites and, as a response, changes in Th17 cell population in Peyer’s patches and T cell TNF production (Li J.-Y. et al., 2016).

#### 6.2.2 Androgens

Males who lose their androgens experience a loss in bone mass as well as an increase in osteoclast-mediated bone resorption. The effect of androgens in osteoclasts is controversial. Androgens inhibited osteoclastogenesis in cultured bone marrow macrophages (BMMs) or RAW264.7 monocyte-macrophage cells, according to two papers (Huber et al., 2001; Steffens et al., 2015). Neither androgenson stromal cells nor osteoblast-lineage cells were responsible for this effect. Another study employing human CD14+ peripheral blood monocytes discovered that androgens have direct and dose-dependent effects on osteoclast production *in vitro* (Michael et al., 2005). Deletion of the androgen receptor (AR), specifically in osteoclasts, showed no effect on *in vivo* osteoclast surface or bone mass, according to a more recent study (Sinnesael et al., 2015). The AR was likewise shown to have relatively low expression in osteoclasts by these researchers. A second group selectively eliminated AR in either mesenchymal or myeloid cells in mice, and discovered that mice with AR deleted in mesenchymal cells had a rapid turnover, osteopenic trabecular bone phenotype (Ucer et al., 2015). Mesenchymal cells lacking the AR were also resistant to trabecular bone loss after orchiectomy. Surprisingly, neither of these animals (mesenchymal or myeloid AR deletion) had a cortical bone phenotype, and both models lost the same amount of cortical bone when orchiectomy was performed (Ucer et al., 2015). As a result, it appears that the regulation of cortical bone loss by androgen deficiency is independent of AR expression in mesenchymal or myeloid cells.

#### 6.2.3 Other Factors

Sexual dimorphism also exists in oral bacterial infections of alveolar bone loss. Compared to women, males have a strong inflammatory response to bacterial infection, resulting in increased inflammatory microenvironment, reduced pathogenic bacteria clearance and increased osteoclast-driven bone loss in response to differential expression of key chemokines (Valerio et al., 2017). Additionally, tungsten has been proven to increase sex-specific osteoclast differentiation in murine bone according to recent research (Chou et al., 2021). Keap1 is a negative controller of the transcription factor Nrf2 for its activity (Yin et al., 2020). The Keap1/Nrf2 signaling pathway has been considered as a master regulator of cytoprotective genes, and exists in many cell types including osteoblasts and osteoclasts (Yin et al., 2020). New finding demonstrates moderating Nrf2 activation by genetic disruption of keap1 has sex-specific effects on bone mass in mice (Yin et al., 2020). Protein kinase C delta (PKC-δ) functions as an important regulator in bone metabolism and conditional knockout of PKC-δ in osteoclasts favors bone mass accrual in males due to decreased osteoclast function (Li S. et al., 2020). Methylphenidate is the most prescribed psychostimulant for ADHD patients, which also participates in the sex-associated behavior changes of osteoclast (Uddin et al., 2018). Methylphenidate regulation of osteoclasts in a dose- and sex-dependent manner adversely affects skeletal mechanical integrity (Uddin et al., 2018).

### 6.3 Aging and Sex Steroid Deficiency Have Independent Effects on Osteoclasts

Aging and sex steroid deficiency have independent effects on osteoclasts (Ucer et al., 2017). The effects of sex steroid deficiency and aging on the osteoclasts, according to a new finding, are independent and come from different pathways (Ucer et al., 2017). In the former, estrogen deficiency results in increased osteoclastogenesis, a prevailing mechanism of cortical bone loss in both genders; the mechanism behind this is likely mediated by mesenchymal/stromal cell-derived SDF1 (Ucer et al., 2017). Loss of cortical bone with aging is largely attributed to decreased osteoblastogenesis caused by increased levels of H_2_O_2_, combined with increased osteoclastogenesis caused by aging mechanisms independent of estrogen deficiency (Ucer et al., 2017).

## 7 Conclusion and Future Perspectives

Osteoclasts, as an important component of the bone microenvironment, have always played an irreplaceable role in bone homeostasis. Abnormalities in osteoclast function can lead to abnormal bone resorption. If osteoclasts are hyperfunctional, they can cause degenerative bone diseases such as osteoporosis and osteoarthritis; if they are dysfunctional or declining, they can cause osteosclerosis. Drugs for bone-related diseases affect the process of bone resorption by osteoclasts in three main ways: differentiation, function, and apoptosis. Therefore, we summarize the biological characteristics of osteoclasts in terms of differentiation, apoptosis, behavior changes, and coupling signals with osteoblasts based on previous studies, in this review. Although we have a more comprehensive understanding of osteoclasts, we still do not know the effects of various modulators on osteoclast behavior in the systemic as well as in the local environment and their mechanisms of action. Additionally, we still have a lot to learn about the processes that control osteoclast sexual dimorphic responses. Research of this phenomena are essential because they can shed light on the pathophysiology of metabolic bone disorders like osteoporosis and how individuals respond to treatment. The identification of gene targets by understanding these mechanisms may lead to more effective treatments for metabolic diseases of the skeleton. As for coupling signals between osteoclast and osteoblast, rather than simply identifying potential coupling factors, it is time to move on to the next phase. It is imperative that we spend time understanding the kinds of mechanisms that drive the remodeling process, and identify the aspects of those mechanisms that can be used to intervene in human skeletal disorders. Furthermore, we think that interactions existing among macrophages, osteoclasts and osteoblasts contribute to maintaining bone homeostasis. Therefore, we believe that pathological connections among these cells in disease states and their negative mechanisms will be a new field for further exploration.
